# Neurotrophic effects of dental pulp stem cells on trigeminal neuronal cells

**DOI:** 10.1038/s41598-020-76684-0

**Published:** 2020-11-12

**Authors:** Nessma Sultan, Laila E. Amin, Ahmed R. Zaher, Mohammed E. Grawish, Ben A. Scheven

**Affiliations:** 1 School of Dentistry, Oral Biology, College of Medical and Dental Sciences, University of Birmingham, Birmingham, UK; 2 Department of Oral Biology, Faculty of Dentistry, Mansoura University, Mansoura, Egypt; 3 Faculty of Dentistry, Horus University, New Damietta, Egypt; 4 Department of Oral Biology, Faculty of Oral and Dental Medicine, Delta University for Science and Technology, Mansoura, Egypt

**Keywords:** Neuroscience, Stem cells

## Abstract

Evidence indicates that dental pulp stem cells (DPSC) secrete neurotrophic factors which play an important role in neurogenesis, neural maintenance and repair. In this study we investigated the trophic potential of DPSC-derived conditioned medium (CM) to protect and regenerate isolated primary trigeminal ganglion neuronal cells (TGNC). DPSC and TGNC were harvested by enzymatic digestion from Wister-Hann rats. CM was collected from 72 h serum-free DPSC cultures and neurotrophic factors; nerve growth factor (NGF), brain-derived neurotrophic factor (BDNF), neurotrophin-3 (NT-3), and glial cell line-derived neurotrophic factor (GDNF) were analysed by specific enzyme-linked immunosorbent assays (ELISAs). Primary co-cultures of DPSC and TGNC were established to evaluate the paracrine effects of DPSC. In comparison, NGF was used to evaluate its neurotrophic and neuritogenic effect on TGNC. Immunocytochemistry was performed to detect the neuronal-markers; neuronal nuclei (NeuN), microtubule-associated protein-2 (MAP-2) and βIII-tubulin. Quantitative real time polymerase chain reaction (qRT-PCR) was used to analyse neuronal-associated gene expression of NeuN, MAP-2, βIII-tubulin in addition to growth-associated protein-43 (GAP-43), Synapsin-I and thermo-sensitive transient receptor potential vanilloid channel-1 (TRPV1). DPSC-CM contained significant levels of NGF, BDNF, NT-3 and GDNF. DPSC and DPSC-CM significantly enhanced TGNC survival with extensive neurite outgrowth and branching as evaluated by immunocytochemistry of neuronal markers. DPSC-CM was more effective in stimulating TGNC survival than co-cultures or NGF treated culture. In comparison to controls, DPSC-CM significantly upregulated gene expression of several neuronal markers as well as TRPV1. This study demonstrated that DPSC-derived factors promoted survival and regeneration of isolated TGNC and may be considered as cell-free therapy for TG nerve repair.

## Introduction

The degree and severity of peripheral nerve injuries vary widely. Severe insults to peripheral nerves may lead to lifelong neurological deficits, including failure of reinnervation and the development of chronic pain. Upon damage, neurons regenerate their axons, and Schwann cells within the injured nerve assist axon regeneration and remyelinate the large axons^1^. Despite this capacity for repair, functional recovery after nerve injuries is still suboptimal in adult mammals, and the outcomes vary widely depending on the degree and severity of the injuries^2^.

The trigeminal ganglion (TG) occupies Meckel’s cave in the dura mater and has three nerve branches, i.e., the ophthalmic, maxillary and mandibular nerves, which supply sensation to key craniofacial structures, including the eyes, tongue and dental tissues^3^. Trigeminal nerve injuries arise primarily from dental procedures and cause significant neurosensory deficits and deleterious effects on several motor and sensory functions that range from chronic and burning pain to paraesthesia and chewing difficulties. Management of such injuries usually occurs through microsurgical repair of a damaged nerve with end-to-end tensionless anastomosis or nerve grafting though overall recovery rates vary and are often inadequate^4^.

A series of cellular events are triggered in response to nerve injury, including signalling from damaged sites, attraction of macrophages, enhanced synthesis of neurotrophins, and regeneration of nerve fibres^5–7^. For the regeneration of damaged or degenerating neurons, the maintenance of neuronal survival and induction of neuronal differentiation are essential processes. Neurite outgrowth is an early process during neuronal differentiation and regeneration and its dynamics is a fundamental process for establishing and maintaining the functional nervous system^8,9^.

Neurons are linked together with neurites, including axons and dendrites, forming a complex neuronal network that allows neuronal communication to regulate various body functions. Different treatment options have been used to promote neuroprotection and neurite outgrowth, one of which is exposure to neurotrophic factors, which are signalling proteins that regulate many aspects of neuronal development, maintenance and survival^8–10^. Nerve growth factor (NGF) is a primary key neurotrophic factor required for neuronal survival and phenotype maintenance^11–14^ Upon binding to its cell surface receptors; TrkA and P75NTR, NGF-receptor complexes initiate the transmission of extracellular cues to intracellular signalling pathways and eventually turn on genes required for neuronal differentiation.

Mesenchymal stem cells (MSC) have become a popular area of interest for their potential in cell-based therapy for treating a range of conditions, including nerve injuries^15,16^. Dental pulp stem cells (DPSC) are cranial neural crest derived MSC present in dental pulp and exhibit multipotent differentiation and a self-renewal ability. DPSC also express immunomodulatory factors that stimulate the formation of blood vessels and enhance the regeneration and repair of injured nerves. The neuroprotective, neurotrophic, angiogenic, and immunomodulatory properties of DPSC make them an attractive cell source for neurodegenerative therapeutic applications^17^. The remarkable mechanism of action after their transplantation is likely to be paracrine-mediated, with secretion of substantial levels of neurotrophic factors coordinated for neuronal survival and axonal regeneration^18^. DPSC secretomes may be harnessed as a cell-free therapeutic tool that reduces the risks associated with engraftment of stem cells, such as the possibility of immune reactions and the development of ectopic tissue^19,20^. In vitro, secretomes are found in the conditioned medium (CM) of cultured cells. It is widely recognized that CM contains a wide range of bioactive secreted factors that may have a pivotal role in regenerative medicine^21^.

In an optic nerve crush experiment, DPSC were shown to protect rat ganglion cells from death and to promote optic nerve regeneration. Various factors were found to be involved in the neuronal effect of DPSC, including NGF, BDNF and NT-3^22,23^. In this study, we investigated the effects of DPSC on primary TGNC, their survival/rescue and neuritogenesis after axotomy of the three main branches of the TG. The study compared the efficiency of secreted factors detected in DPSC-CM with those of DPSC/TGNC co-cultures comparing between the established cell-based therapy using DPSC and cell-free therapy using DPSC-CM.

## Materials and methods

DPSC and TGNC were isolated from 100 to 150 g, 4–6 w old male Wister-Hann rats (University of Aston, Pharmaceutical Sciences Animal House, Birmingham, UK). All experiments were conducted on dead animals that were not sacrificed for the purpose of the study. All the methods were carried out in accordance with university guidelines and regulations. All plastic-ware used was supplied by Thermo Fischer Scientific, UK, and most of the materials used were supplied by Sigma Aldrich, UK, unless stated otherwise.

### Isolation of DPSC

DPSC were isolated consecutively from 15 rats (5 rats/experiment) via enzymatic digestion according to routine methods established in our laboratory^24,25^. Isolated pulp tissues from rat incisors were pooled together on a cooled glass plate and fragmented. The combined minced tissue was transferred to a Falcon tube for enzymatic digestion with 4 mg/mL collagenase I (Merck, Millipore, UK) for 30 min at 37 °C with 5% CO_2_. Digestion was interrupted by the addition of complete growth medium (alpha-minimum essential medium (α-MEM, Biosera, UK) supplemented with 100 μg/ml penicillin, 100 μg/ml streptomycin, 2 mM L-glutamine and 10% foetal bovine serum (FBS)). The suspension was poured through a 70 µm cell sieve and collected in a 50 ml Falcon tube, which was centrifuged (Jouan, UK) at 1200 rpm for 3 min. The isolated cells were cultured in α-MEM supplemented with 10% FBS in a T75 flask and were incubated at 37 °C with 5% CO_2_ environment. Upon reaching 70–80% confluence, the cells were subcultured following treatment with 0.25% trypsin–EDTA for 10 min at 37 °C and then were passaged to new culture flasks or used in experiments. DPSC from the 2nd to 5th passages were used in this study. Cells were collected for phenotypic characterization and differentiation induction at the 3rd passage.

### Characterization of DPSC using semi-quantitative PCR (sqRT-PCR)

To characterize the adherent cells, sqRT-PCR was used to evaluate surface marker expression. Briefly, total RNA was prepared from confluent DPSC cultures by using the RNeasy Mini Kit (Qiagen, UK) consisting of RLT lysis buffer and washing buffers (RW1 and RPE). Extracted RNA was used to generate cDNA using a Tetro cDNA synthesis kit (Bioline, UK). The cDNA was amplified for sqRT-PCR by using REDTaq ready reaction mix. The intensity was normalized to expression level of the endogenous reference gene GAPDH. The sequences of the Rattus novergicus primers are illustrated in Table 1.

**Table 1:** Specific forward and reverse primer sequences used in sqRT-PCR for the different gene.

Genes	Primer sequences (5–3′)	
	Forward	Reverse
CD105	F-TTCAGCTTTCTCCTCCGTGT	R-TGTGGTTGGTACTGCTGCTC
CD90	F-AGCTCTTTGATCTGCCGTGT	R-CTGCAGGCAATCCAATTTTT
CD29	F-ATCATGCAGGTTGCAGTTTG	R-CGTGGAAAACACCAGCAGT
CD14	F-GTTGGGCGAGAAAGGACTGA	R-GCTCCAGCCCAGTGAAAGAT
CD45	F-AGCTACCCCTCAAACGAAGC	R-TGTGAGTCCCTGGTGGTACA
Nestin	F-CAT TTA GAT GCT CCC CAG GA	R-AAT CCC CAT CTA CCC CAC TC
NANOG	F-TATCGTTTTGAGGGGTGAGG	R-CAGCTGGCACTGGTTTATCA
SOX2	F-TCCAGTCAAGCCCCACATC	R-TCCGAGTCACCCTTCCCA
LPL	F-GTCACCAGCATCCCCATTAT	R-TTCCGGATAAAACGTTCTCG
ALK-phosphatase	F-CTC CGG ATC CTG ACA AAG AA	R-ACG TGG GGG ATG TAG TTC TG
OPN	F-AAG CCT GAC CCA TCT CAG AA	R-GCA ACT GGG ATG ACC TTG AT
NeuN	F-GTGCTGACCTCTATGGTGGA	R-TGTGTACACCCTGCCGTAAC
GFAP	F-AGGCTAATGACTATCGCCGC	R-TCCTTAATGACCTCGCCATCC
GAPDH	F-CCCATCACCATCTTCCAGGAGC	R-CCAGTGAGCTTCCCGTTCAGC

### Osteogenic and adipogenic differentiation of DPSC

To verify the mesenchymal properties of DPSC, we tested their ability to generate either osteoblasts or adipocytes using appropriate media and culture conditions. For in-vitro osteogenic and adipogenic differentiation, when the cultures reached 70% confluence, osteogenic or adipogenic medium was added for 21 days to determine the differentiation potential of the cells into classical mesodermal lineages. For each differentiation assay, 4 plates were used (2 for RNA isolation and 2 for staining).

For osteogenic induction, DPSC were cultured in α-MEM medium supplemented with 100 nM dexamethasone, 10 mM sodium-β glycerol phosphate and 50 nM ascorbic acid, and they received three weekly medium changes. Cell differentiation was assessed by alizarin red staining, as an indicator of extracellular matrix calcification. For alizarin red staining, cultures were fixed in 10% neutral buffered formaldehyde (Leica Biosystems, UK) at room temperature for 15 min. Cultures were washed twice with Dulbecco’s PBS (DPBS) without Ca^2+^/Mg^2+^, and the cells were incubated in 1% alizarin red stain for 20 min at room temperature. Then, the wells were washed with distilled water four to five times until the media became clear. Finally, the cells were visualized and analysed under a phase-contrast microscope.

For adipogenic induction, DPSC were cultured in α-MEM medium supplemented with 100 nM dexamethasone, 200 μM indomethacin and 0.5 mM isobutyl-methyl xanthine (IBMX), and they received three weekly medium changes. Oil red O (ORO) staining (Merk, cat. No. O1391-250ML), which indicates intracellular lipid accumulation, was performed following the manufacturer’s instructions. Cells were washed with PBS and fixed in 10% formalin for 1 h. After washing with 60% isopropanol, cells were stained with ORO solution in 60% isopropanol for 5 min, rinsed with deionized water, and treated to two consecutive washes in PBS. Finally, the cells were visualized and analysed under a phase-contrast microscope. Total RNA was prepared from undifferentiated and differentiated cultures using the same technique described earlier. Primers used in analysis of mineralization and lipid formation are illustrated in Table 1.

### Collection of DPSC-CM

When DPSC reached 70–80% confluence, they were used for the preparation and collection of CM. Growth medium was removed, cells were washed twice with PBS, and the culture medium was replaced with serum-free α-MEM. After 72 h of incubation, the culture medium was collected and centrifuged for 5 min at 1500 rpm at 4 °C. The supernatants were collected and centrifuged for another 3 min at 3000 rpm at 4 °C. The resulting supernatants (denoted as DPSC-CM) were passed through a 0.22-μm filter unit (Merck, Millipore, Ireland) and stored in small cryo-tubes at − 80 °C for further experiments^26^.

### Quantification of secreted neurotrophic factors

Total protein content was quantified by a bicinchoninic acid assay (BCA) kit (The Thermo Scientific, Pierce BCA). Enzyme-linked immunosorbent assays (ELISAs) were performed according to the manufacturer’s instructions on the pooled DPSC-CM to determine the concentrations of rat NGF and BDNF (R&D Systems, Biotechne, UK), GDNF (Boster Picokine, CA, USA, EK0935), NT-3 (Fine Test, MD, USA, ER0055), and ciliary neurotrophic factor (CNTF, Thermo Scientific, UK, ERCNTF). The pooled DPSC-CM was analysed in triplicate and the absorbance was measured at 450 nm using a Tecan plate reader. The quantities of factors (pg/ml) were calculated based on a standard curve.

### Co-culture experiments

DPSC were seeded at 10^5^ cells/200 µl into polyester Transwell inserts with 1 µm pore size in 24-well plates (Merck, Millipore, UK). DPSC were seeded on top of the membrane in complete growth medium with 10% FBS. After 24 h, the culture medium was replaced with serum-free α-MEM, and inserts were placed over the lower chamber containing the neuronal cell cultures.

### Isolation of primary trigeminal ganglion neuronal cells (TGNC)

Isolation and culture of the rat TGNC was carried out in accordance with the method described by Malin et al.^27^. The entire brain was removed from the skull, and the TGs were identified at the base of the cranium. Spring scissors were used to cut the three main branches (ophthalmic, maxillary and mandibular) of the TGs at their connection to the brain. The posterior end of the ganglion was grasped and lifted while the connective tissue was clipped to free the TGs from the dura mater. Both ganglia were placed into 35 mm culture dishes containing 2 ml Hank's balanced salt solution without Ca^2+^/ Mg^2+^ and were kept on ice. TGs were enzymatically digested for 1 h with 4 mg/ml collagenase I that was prewarmed at 37 °C for 20 min before use. Ten units of DNase I (Roche, Indianapolis, IN) was added in the last 10 min of enzymatic digestion. The tissue digest was fractionated on a Percoll gradient to separate myelin and nerve debris from the TGNC; in a tissue culture hood, 1.1 ml of Percoll was added to 2.9 ml of warm complete medium in a 15 ml tube (28% Percoll). In another tube, 0.5 ml of Percoll was added to 3.5 ml of warm complete medium (12.5% Percoll). Then, 12.5% Percoll was gently layered over 28% Percoll. The cell suspension was added to the Percoll gradient and centrifuged for 10 min at 3000 rpm. Cells were pelleted at the bottom of the tube, while nerve debris was evident at the Percoll interface. The supernatants were removed, and the cell pellet was resuspended in 600 µl of complete medium. 100 µl cell suspension was pipetted carefully onto collagen coated coverslips (Electron Microscopy Science, UK), placed in 6-well plates and kept in the incubator for 2 h for initial attachment before flooding each well with 1 ml of warm DMEM/F12 + 10% FBS. The medium was changed after 48 h and then every second day.

For each experiment, TGNC (5 × 10^3 ^cells/cm^2^) were cultured in DMEM/F12 without FBS as a negative control or in the presence of 50 ng/ml NGF^28^ as a positive control; alternatively, TGNC were cultured with serum-free fresh medium combined with 50% DPSC-CM, or they were co-cultured with DPSC for 4 days.

### Characterization of TGNC culture

TGNC cultured in complete growth medium (DMEM/F12 + 10% FBS) were examined at 4, 10 and 21 days under phase-contrast microscopy, and then were used for laser confocal immunocytochemistry and sqRT-PCR analysis of mature neuronal markers NeuN and the glial cell marker GFAP. The average number of NeuN and GFAP immunopositive cells was counted using the ImageJ cell counter plugin from 30 to 35 images/time point for each marker, obtained from 3 independent experiments.

### Cell viability assessments

#### MTT viability assay

Cell metabolic activity under different culture conditions was analysed by the colorimetric MTT assay. Briefly, cells were cultured with 50 µl of 5 mg/ml MTT for 4 h, which was followed by dissolution of the resulting formazan crystals in 200 µl of DMSO (Fisher Scientific, UK) containing 6.25% (v/v) 0.1 M NaOH. The absorbance was measured using a microplate ELx800 plate reader (Biotek Instruments, INC, UK) at a wavelength of 570 nm.

#### Live and dead cell assay

To distinguish between live and dead TGNC, cultures were stained with calcein-AM and ethidium homodimer-1 (EthD-1) according to the manufacturer’s instructions (Live/dead assay; Invitrogen, Thermo Fisher Scientific, USA). The fluorescence in the experimental and control cell samples was measured using excitation and emission filters (calcein-AM = 494/517 nm and EthD-1 = 528/617 nm).

### Neurite outgrowth assessments

#### Image analysis of neurites

Analysis was based on imaging of cells using confocal microscopy followed by manual tracing of neurite length on MAP-2 immunostained images using a simple neurite tracer plugin. The number of surviving neuronal cells in NeuN immunostained images were counted using ImageJ software. The data were obtained from 3 independent experiments with 10–15 replicates for each group/experiment. Analysis was obtained by combining two fields from a single well, and data are expressed as the average cell number and neurite length in µm.

### Immunocytochemical fluorescence staining

Cultures were fixed with 4% paraformaldehyde (Alfa Aesar, MA, USA) for 10 min and permeabilized with 0.2% Triton X-100 for 10 min at room temperature. After blocking nonspecific binding sites with 3% BSA (Sigma) and 10% goat serum at room temperature for 1 h, cells were incubated overnight at 4 °C with primary antibodies against cytoskeletal marker polyclonal anti-mouse βIII-tubulin (cat# ab7751 at a 1:1000 dilution, Abcam, Cambridge, UK), mature neuronal markers; polyclonal anti-rabbit NeuN (cat# ab128886 at a 1:1000 dilution, Abcam), monoclonal anti-mouse MAP-2 (cat# ab11267 at a 1:500 dilution, Abcam), and glial cell marker polyclonal anti-rabbit GFAP (cat# ab7260 at a 1:1000 dilution, Abcam). Immune reactivity was visualized with secondary conjugate Alexa Flour 488 (cat# ab150113 and ab6717 at a 1:200 dilution, Abcam) and Alexa Flour 555 (cat# ab150078 and ab6786 at a 1:200 dilution, Abcam), which were incubated with the cells for 1 h at room temperature. DAPI was used for nuclear counterstaining. Images were captured using laser confocal microscopy (ZEISS LSM 700).

### Quantitative reverse transcription-polymerase chain reaction (RT-PCR)

Quantitative (qRT-PCR) was performed to measure the mRNA expression levels of neuronal markers in TGNC cultures under different culture conditions. Briefly, total RNA was extracted from TGNC cultures by using the RNeasy Mini Kit (Qiagen, UK) The cDNA template was used in qRT-PCR reaction by using SYBER green reagents (Roche Diagnostics, USA). The specific forward and reverse primers used in qRT-PCR are shown in Table 2. Expression levels were obtained from Cp values for each sample by employing the fit points method as computed by the Light Cycler 480 software (Roche Diagnostics, USA), and GAPDH was used as housekeeper control gene.

**Table 2:** Specific forward and reverse primer sequences used in qRT-PCR for the different genes.

Genes	Primer sequences (5′-3′)	
	Forward	Reverse
NeuN	F-CATGACCCTCTACACGCC	R-TGGAGTTGCTGGCTATCTGT
MAP-2	F-GATCAACGGAGAGCTGACCT	R-TTGGGCCTCCTTCTCTTGTT
BIII-tubulin	F-ATGAGGGAGATCGTGCACA	R-CACGACATCCAGGACTGAGT
GAP-43	F-GTTGAAAAGAATGATGAGGACCA	R-GCATCACCCTTCTTCTCGT
Synapsin-I	F-CCCAGATGGTTCGACTACAC	R-GGGTATGTTGTGCTGCTGAG
TRPV1	F-TCCAGTCAAGCCCCACATC	R-TCCGAGTCACCCTTCCCA
GAPDH	F-CTACCCACGGCAAGTTCAAC	R-CCAGTAGACTCCACGACATAC

### Statistical analysis

Shapiro–Wilk normality test was used first to determine if the data were normally distributed. One-way analysis of variance (ANOVA) followed by a post hoc Tuckey test (IBM-SPSS 26 software) was used to determine significant differences between experimental groups with normally distributed data (Figs. 2, 3, 4, 5). Statistical significance was set as *p $\le$ 0.05, **p $\le$ 0.01, and ***p $\le$ 0.001.

Non-parametric Kruskal–Wallis followed by Mann–Whitney tests were used to analyse the RT-PCR data. Like most non-parametric tests, it was performed on ranked data, so the measurement observations were converted to their ranks in the overall data set and are shown in tables. *p* $\le$ 0.05 shows it is significant.

### Ethical approval

The authors declare no conflicts of interest with respect to the authorship and/or publication of this article.

## Results

### Characterization of DPSC culture

**Figure 1:**
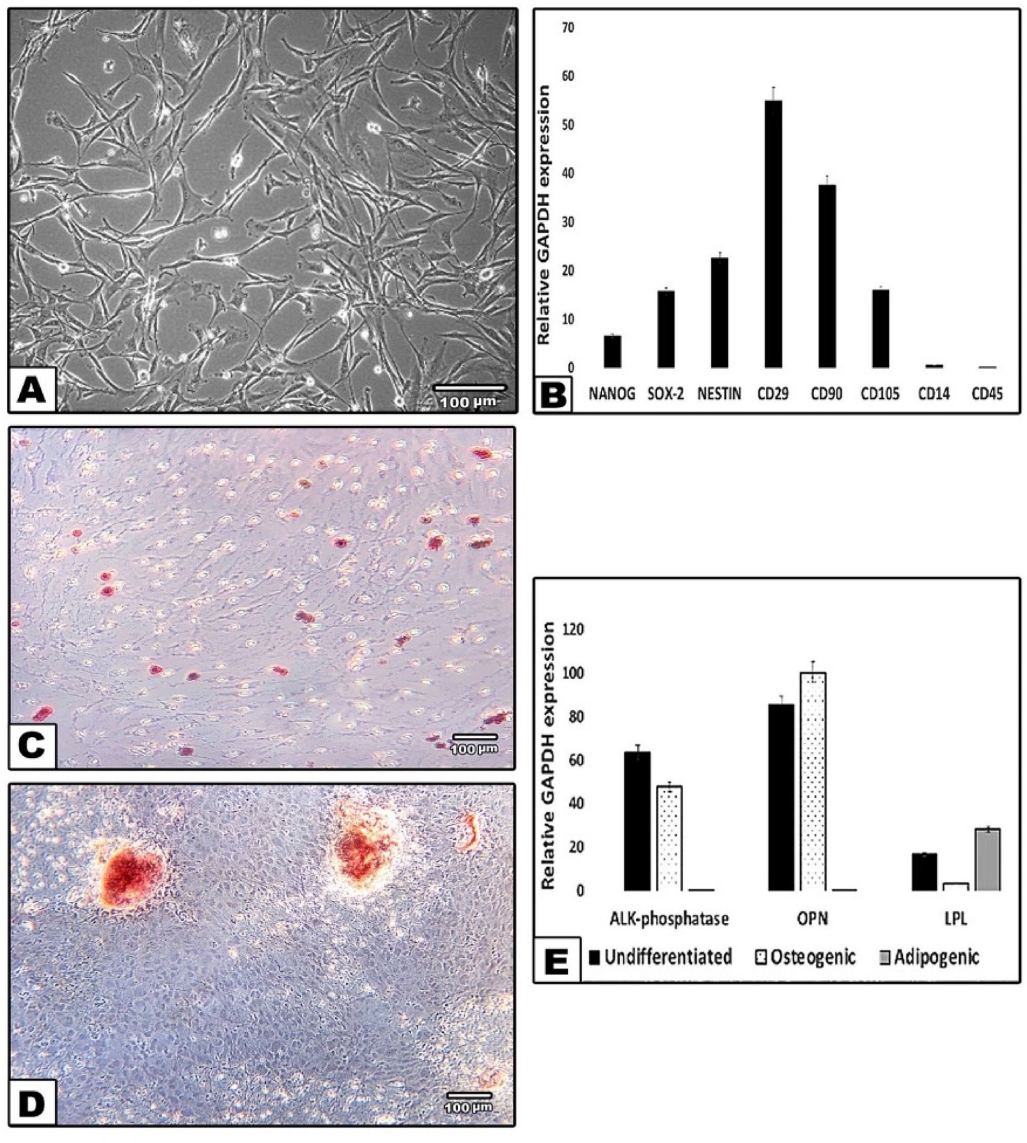
Characterization of DPSC cultures. (**A**) Representative phase-contrast microscopic image of DPSC at passage 3 displays a spindle-like morphology with relatively thin processes extending from the cell body. (**B**) Relative expression profiles of Nanog, Sox2, Nestin, CD29, CD90, CD105, CD14 and CD45 in DPSC in relation to the expression of a housekeeping gene, GAPDH. (**C**) Differentiation along adipogenic lineage (Oil red O), (**D**) Osteogenic lineage (alizarin red). Scale bar: 100 μm. (**E**) SqRT-PCR confirming the osteogenic and adipogenic differentiation of DPSC by the expression of mineralization markers; alkaline-phosphatase (ALP), osteopontin (OPN) and adipogenic marker; lipoprotein lipase (LPL) in the three tested groups; undifferentiated control, osteogenic and adipogenic. GAPDH is a housekeeping control.

DPSC isolated from dissociated dental pulp adhered to plastic surfaces, and cells generally exhibited a fibroblast-like morphology (Fig. 1A). Cells at the 3rd passage were collected for phenotypic characterization and differentiation induction. The results showed that DPSC expressed MSC markers: CD105, CD90, and CD29 and very low levels of specific hematopoietic cell markers; CD14 and CD45. The pluripotent markers; Nanog and SOX2 and the neural marker Nestin were positively expressed (Fig. 1B). The cultured cells were successfully differentiated into osteogenic and adipogenic lineages, which were analysed by alizarin red and ORO staining, respectively (Fig. 1C,D). Undifferentiated and differentiated DPSC were analysed by sqRT-PCR, and the results showed positive expression of mineralisation markers; alkaline phosphatase (ALP) and osteopontin (OSP) as well as the adipogenic marker lipoprotein lipase (LPL) (Fig. 1E).

### Characterization of TGNC culture

**Figure 2:**
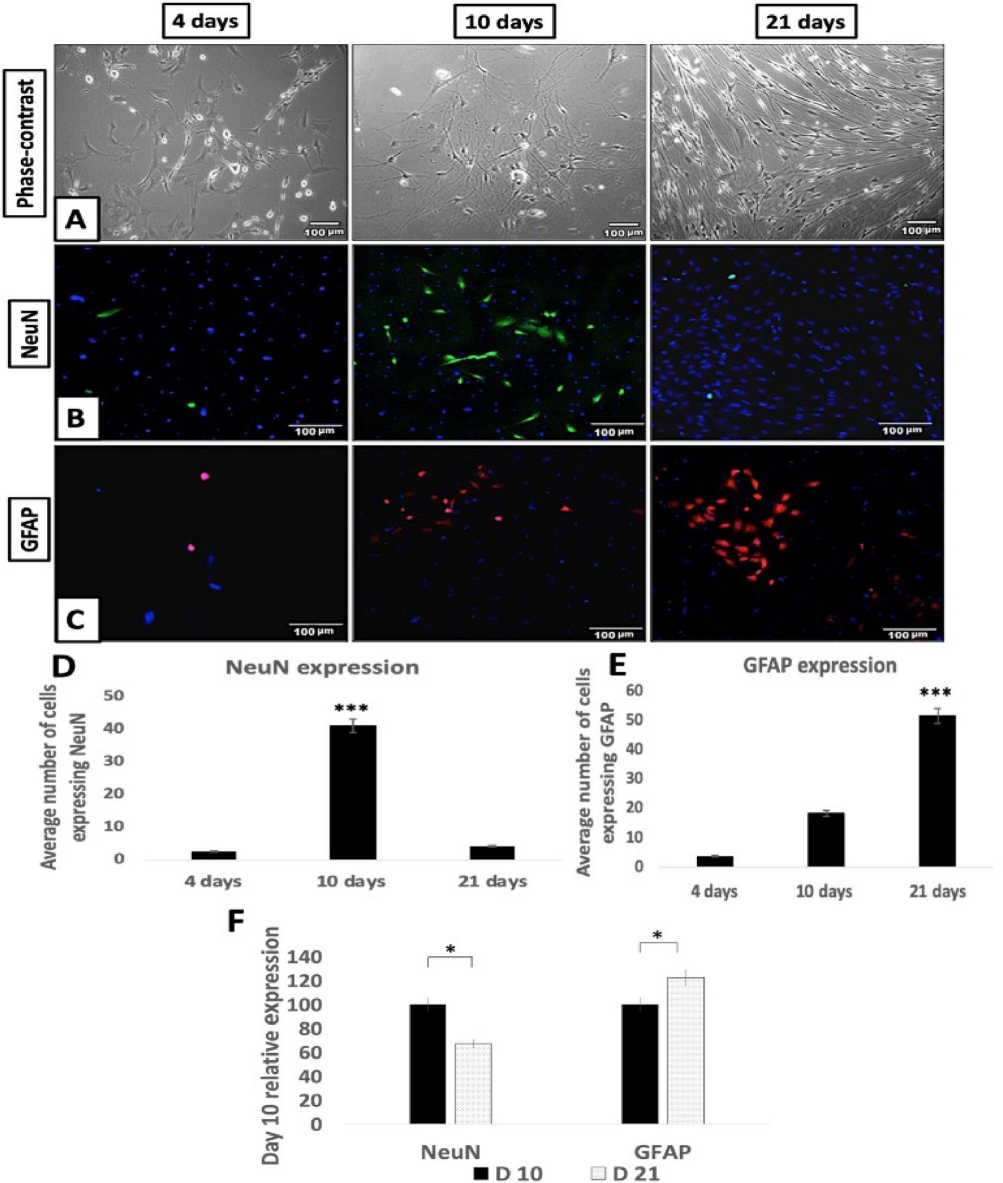
Characterization of isolated TGNC: (**A**) Phase-contrast images, (**B**) NeuN & (**C**) GFAP immunofluorescence expression. Morphology of primary isolated TGNC was followed at 4, 10 and 21 days in complete growth medium. The phase-contrast microscopic images showing that the neuronal cell bodies are rounded and phase bright, while the putative glial cells are spindle in shape. The expression of NeuN and GFAP in TGNC fresh culture was in green and red, respectively, while DAPI was used to stain nuclei in blue. Scale bar: 100 µm. (**D,E**) Quantitative analysis of NeuN and GFAP immunopositive cells using ImageJ analysis, the results are the average number of stained nuclei for each marker. GFAP gradually increased over time; NeuN-positive cells increased over the first 10 days, were decreased by day 21. Data are presented as mean ± SEM, from 3 independent experiments with 10 replicates for each group/experiment. (**F**) SqRT-PCR analysis of NeuN and GFAP using GAPDH as housekeeping control gene. The data showing reduction in the expression of NeuN mRNA with a concomitant increase in GFAP mRNA gene expression after 21 days. Data are presented as a relative percentage to day 10.

Phase-contrast microscopy was used to examine the morphology of isolated TGNC after 4, 10 and 21 days in culture (Fig. 2). These observations allowed interpretation of morphological development during 21 days of culture. Three stages were noted during culture: from 0 to 4 days, cells were scattered over the bottom of the flask as individual cells, and single neurons were sometimes detected in this stage and characterized by having a large cell body surrounded by a bright halo. At 4–10 days, a clear increase in the cell number of neuronal-like cells was detected. Putative glial cells had a spindle shape with a long process and gathered in clusters across the bottom of the flask. At 10–21 days, most of the cells in culture had gained a glial spindle shaped appearance and were aligned in the same direction.

Immunofluorescence staining was used to examine the expression of the mature neuronal marker NeuN and the glial marker GFAP in the cultures over time (4 to 21 days). The cultures displayed distinct immunopositivity for NeuN and GFAP cells. Image analysis-assisted counting indicated a clear increase in the number of cells expressing GFAP in response to culture time however, NeuN immunopositive cells first increased in number and then were reduced over time after prolonged culture.

These observations were confirmed by sqRT-PCR showing gene expression for the mature neuronal marker NeuN and glial marker GFAP. NeuN gene expression was reduced at day 21 with a concomitant and significant increase in GFAP mRNA expression. These findings suggest an initial loss of neuronal cells due to the isolation procedure which involves axotomy, and then there is a gradual increase in neuronal cells over the first 10 days of culture. Then, the loss of neuronal cells occurred due to the absence of trophic support, while there was an increase in the number of glial-like cells during the extended culture period.

### Optimizing the concentration of DPSC-CM

**Figure 3:**
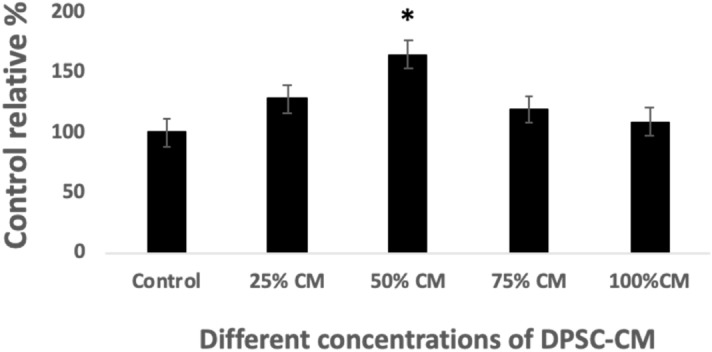
Optimizing the concentration of DPSC-CM. Cytotoxicity study through MTT assay showing the relative percentage of neuronal cell viability exposed to different concentrations of DPSC-CM for 4 days. Control group was set to 100% to be considered as a basal and compared with the values of corresponding exposed samples. Data are presented as mean ± SEM, from 3 independent experiments with 10 replicates for each group/experiment.

The MTT assay was used to assess neuronal cell viability after exposure to different concentrations of DPSC-CM. The data indicated that DPSC-CM optimally promoted cell viability at 50% concentration, while there was a significant reduction in cell viability at 75% and 100% (pure CM) (Fig. 3). Consequently, 50% DPSC-CM was used in subsequent experiments.

### DPSC promote TGNC survival and viability

**Figure 4:**
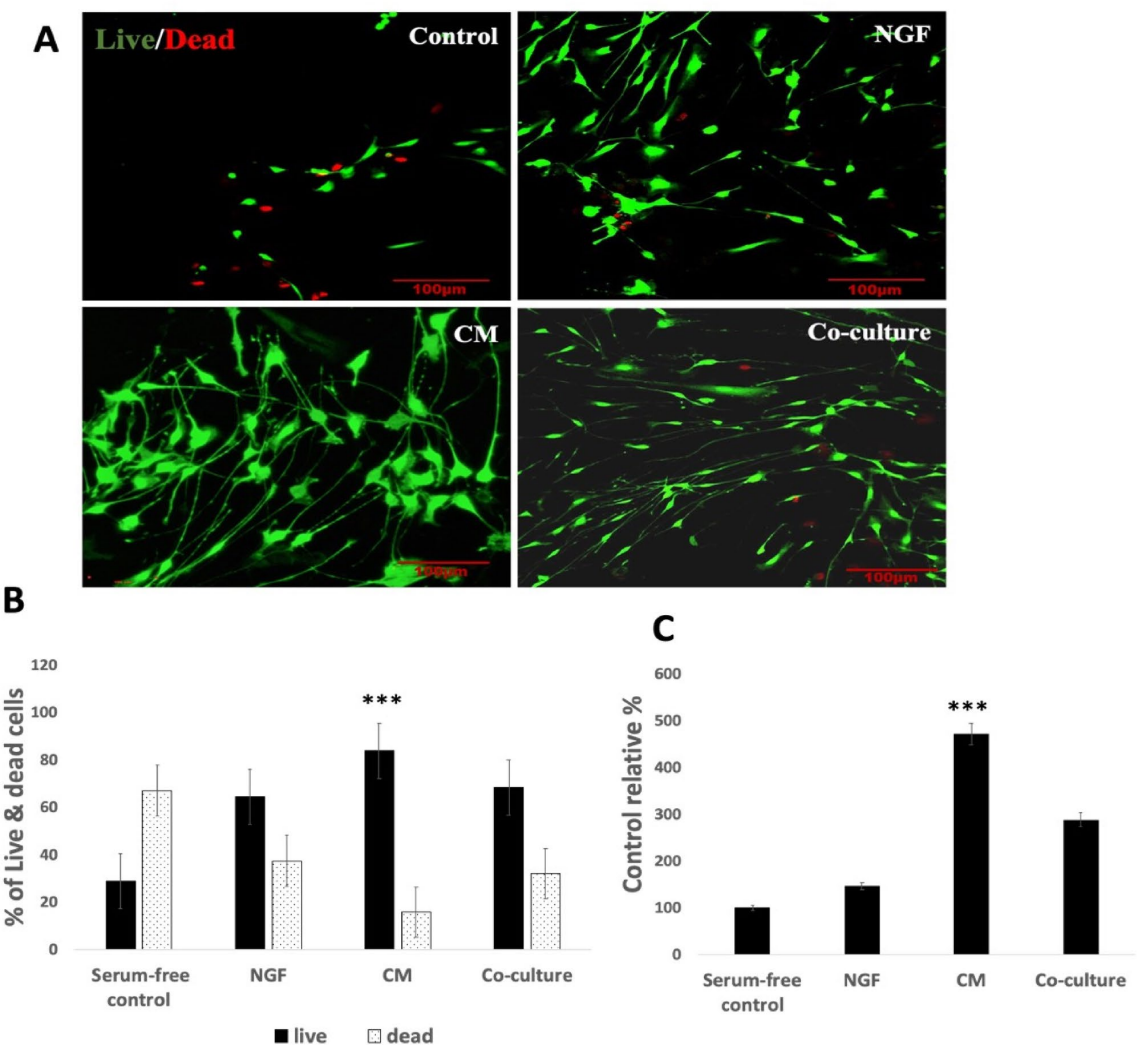
DPSC promote cell survival and decrease cell death. After 4 days exposure period of TGNC to; serum-free DMEM/F12 (control), 50% DPSC-CM, 50 ng/ml NGF or co-cultured with DPSC, the number of viable cells was significantly higher in DPSC-CM treated cultures. (**A**) Laser confocal images of live/dead TGNC assay, scale bar: 100 µm. (**B**) Quantitative live/dead assay results obtained from Tecan fluorescence plate reader. (**C**) Total viable cell number is confirmed by MTT assay, serum-free control was kept at 100% and all other groups were relative to it. Data are presented as mean percentage ± SEM, from 3 independent experiments with 15 replicates for each group/experiment.

The MTT viability data and the live/dead cell assay showed that DPSC-CM significantly increased the number of viable TGNC in comparison with what was observed in the negative and positive controls. In the serum-free control group, 60% of the culture was showing cell death, while only 15% of cell death was detected in the DPSC-CM group. Interestingly, there was a statistically significant difference in the live cell percentage between the CM-treated group and DPSC co-cultured with TGNC, indicating that CM was more potent in providing cell protection than co-cultures (Fig. 4).

### DPSC stimulate neurite outgrowth

**Figure 5:**
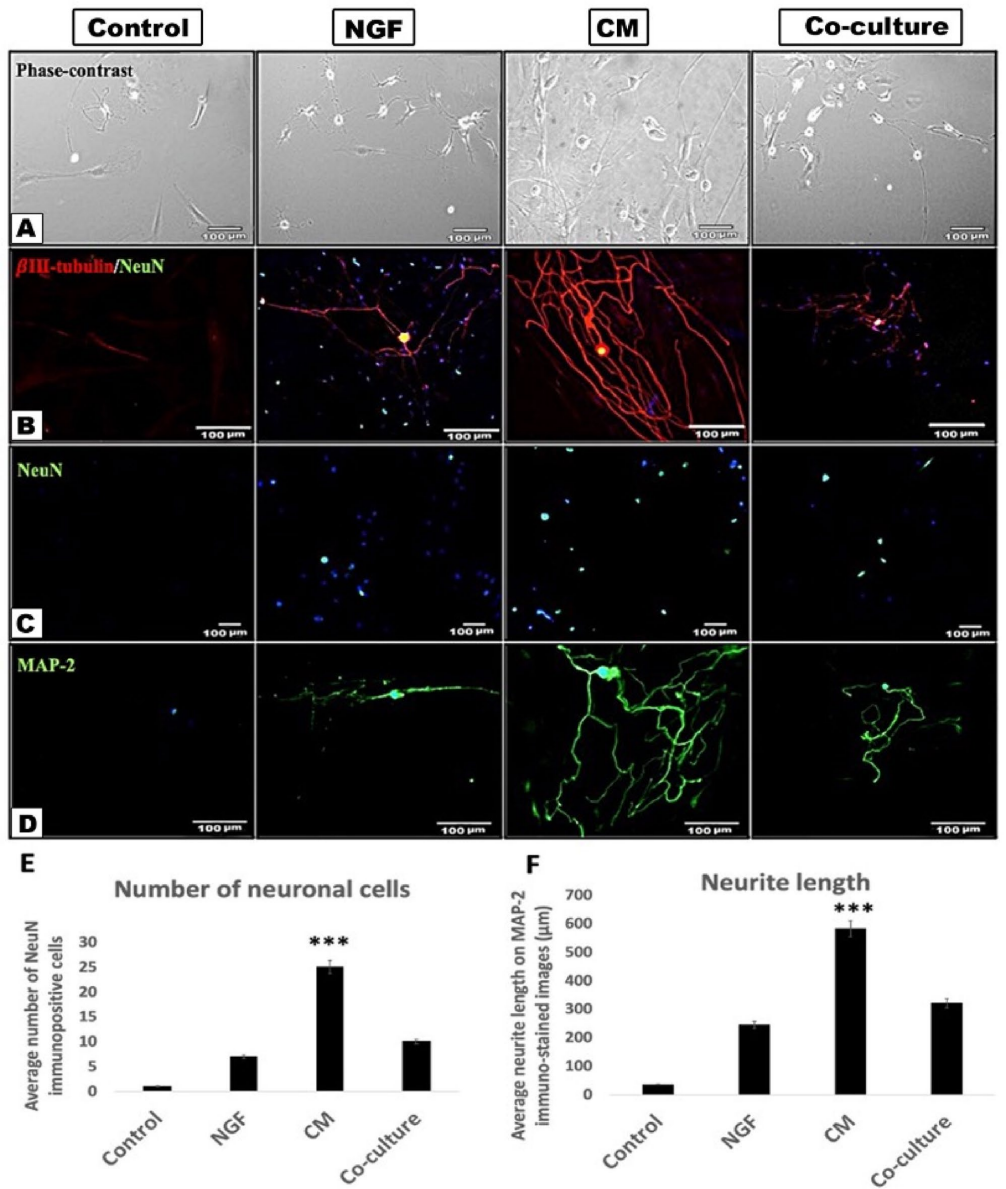
DPSC-CM greatly stimulate TGNC survival and neurite outgrowth. (**A**) Phase-contrast images of TGNC after 4 days exposure period to; serum-free DMEM/F12 (control), 50 ng/ml NGF, 50% DPSC-CM, or co-cultured with DPSC. (**B**) Double immunocytochemistry of cytoskeletal βIII-tubulin outline the cell cytoskeleton (red) and mature neuronal marker NeuN outlines the neuronal nucleus (green). Yellow color in the nucleus because of the overlay between red and green colors. (**C**) NeuN immunocytochemistry showing difference in the number of surviving neurons under different treatment conditions. (**D**) MAP-2 immunocytochemistry clearly stain the neurite processes (green). DAPI (blue) was used as a nuclear staining. (**E**,**F**) Quantification of the average number of neurons and the average neurite length as meaured by ImageJ software on NeuN and MAP-2 immunostained images, respectively. Data are presented as mean ± SEM from 3 independent experiments with 10–15 replicates for each group/experiment. Scale bar: 100 µm.

The morphological changes induced in TGNC upon exposure to different treatment conditions were assessed by morphometric analysis on immunostained images (Fig. 5).

Morphometric analysis of neuronal cells exposed to serum-free DMEM/F12 condition revealed that the number of neurons per field was greatly reduced compared to those in the DPSC-CM treated culture. In the serum-free DMEM/F12 control group, the neurite length was very short and hardly recognized, however, the group treated with DPSC-CM showed a significantly higher number of neurons with extensive neurite outgrowths compared to what was observed in other treatment conditions.

βIII-tubulin/NeuN and MAP-2 immunocytochemistry were used to further outline the difference in the TGNC neurite lengths exposed to different treatments. The immunostaining revealed that the serum-free control group was devoid of any neuronal cells, underscoring the cell loss in this group. However, cells treated with DPSC-CM showed robust neurite outgrowth and branching stained with the mature neuronal marker MAP-2 and cytoskeletal marker βIII-tubulin, which appeared more prominent than what was seen in the co-culture and NGF treated cultures.

NeuN immunostaining revealed a difference in the number of surviving neurons in the treated cultures; serum-free control culture showed a marked reduction in the number of surviving neurons, while there was an increase in the number of NeuN-positive neurons cultured with DPSC-CM.

PCR data indicated that DPSC-CM upregulated neuronal markers in the TG cultures. NeuN and βIII-tubulin were highly expressed in the DPSC-CM group compared to the other groups; however, MAP-2 was similarly expressed in all treated groups, albeit significantly higher than that of the control cultures (*p* < 0.05).

Synapsin-I is an indicator of neuronal synapse formation, and GAP-43 is an important partner in neuronal differentiation, plasticity and regeneration after nerve injury. An increase in synapsin-I was observed in all groups being significantly higher in CM treated cultures than NGF treated culture (*p* = 0.05), while GAP-43 was upregulated in DPSC cultures and downregulated in NGF treated cultures. GAP-43 showed modest expression among DPSC cultures but was significantly higher than that in the NGF treated group (*p* = 0.02).

The TGN conveys sensory inputs, including painful stimuli from tooth pulp and mucosal surfaces. To understand the possible modulatory influence of DPSC-neurotrophic factors on TGN nociception transducing receptors, TRPV1 mRNA gene expression was analysed. The TRPV1 level was significantly upregulated in the DPSC-CM treated group in comparison to the co-culture and NGF cultures (*p* = 0.05), suggesting a contribution of DPSC-CM in stimulation of nociceptive receptors of isolated TGNC (Table 3).

**Table 3:** Comparison of TGNC different gene expression levels after being cultured with; control, 50 ng/ml NGF, 50%DPSC-CM and TGNC/DPSC co-culture using Kruskal–Wallis non-parametric test.

Groups (mean rank)							
Genes	n		Control	NGF	DPSC-CM	Co-culture	*P* value*
NeuN	3		3.50	5.50	13.25	11.75	0.007
		*P* value**		P1 = 0.78	P1 = 0.025 P2 = 0.021	P1 = 0.029 P2 = 0.032 P3 = 0.086	
MAP-2			2.50	13.00	9.75	9.25	0.016
		*P* value**		P1 = 0.014	P1 = 0.029 P2 = 0.21	P1 = 0.029 P2 = 0.21 P3 = 1.90	
BIII-tubulin			4.50	10.00	12.00	7.50	0.130
GAP-43			6.50	2.50	12.00	13.00	0.005
		*P* value**		P1 = 0.06	P1 = 0.031 P2 = 0.021	P1 = 0.023 P2 = 0.020 P3 = 0.14	
Synapsin-I			4.50	6.75	13.25	9.50	0.054
		*P* value**		P1 = 0.34	P1 = 0.01 P2 = 0.057	P1 = 0.05 P2 = 0.06 P3 = 0.14	
TRPV1			3.50	7.00	13.75	7.75	0.051
		*P* value**		P1 = 0.015	P1 = 0.01 P2 = 0.05	P1 = 0.015 P2 = 0.21 P3 = 0.05	

P1: significant *vs* control, P2: significant versus NGF, P3: significant versus DPSC-CM.

* Kruskal–Wallis, ** Mann–Whitney, *P*: Probability.

### Quantification of secreted neurotrophins in DPSC-CM

To further understand the mode of action of DPSC-CM, its protein content was analysed. The total protein concentration in the pooled collected serum-free DPSC-CM was 1800 µg/ml. Using ELISAs, the neurotrophic factors NGF, BDNF, GDNF and NT-3 were detected in the pooled DPSC-CM. High quantities of the NGF and NT-3 were detected (191.6 pg/ml and 161.1 pg/ml, respectively), and BDNF and GDNF amounted to 85.5 pg/ml and 90.2 pg/ml, respectively. Only a trace amount of CNTF could be detected.

## Discussion

In this study, freshly isolated primary TGNC cultures maintained in culture for up to 21 days and were characterized. TGNC and their surrounding putative glia were maintained in culture for up to 10 days without an evident reduction in the immunoreactivity of GFAP and NeuN. NeuN is expressed by neuronal cells and GFAP is expressed by non-myelinating glial cells in the nervous system; thus, they are considered reliable markers and were used in this study^29,30^. After 10 days, it was clear that the relative number of glial cells increased associated with decrease in the number of neuronal cells^31^. Therefore, in this context, the experiments were carried out for 4 days because during extended periods of culture, non-neuronal cells proliferate during this time, consume more nutrients and cover the neuronal cells, making it hard to study growth factor-dependent cell survival, axon outgrowth and basic mechanisms of sensory physiology^27^.

Cell therapy using MSC has emerged as a novel and promising neuroprotective strategy. Accumulating data have demonstrated that DPSC may enhance neuronal survival and neurite outgrowth via a paracrine mechanism, making them good candidates for helping in peripheral nerve repair^22,23,32^. In particular, here, we tried to make a rational comparison between DPSC and their CM, evaluating their neurotrophic and neuritogenic effects on primary isolated TGNC.

In our study, a significant degree of neuroprotection and neurotrophic effects under co-culture conditions was observed in the absence of serum and without cell–cell contact, which indicated that DPSC exert their action via their secretome. These findings correspond with previously published data^32^ that demonstrated the paracrine effects of DPSC on axotomized retinal ganglion cells. In the present study, neuronal survival and neurite length in the culture treated with DPSC-CM were significantly higher than those in the co-culture treated group. This finding suggests that DPSC co-culture failed to provide a rapid protection for the primary neuronal cells than CM, highlighting that the live cell co-cultures had a delayed lag time in producing effective amounts of trophic factors. Thus, the advantage of using CM over co-culture/cell therapy treatment may be attributed to the lag-time required by DPSC to secrete neurotrophic factors, making cell-free therapy based on DPSC secretome superior to cell-based applications using live cells. However, it may argue that cell therapy may have longer lasting effects than cell-free approaches. Palomares et al. compared the capacities of adipose mesenchymal stem cells (ASC) and their CM to recover oxidized SH-SY5Y cell viability and concluded that both treatments had the same capacity to recover the viability of oxidized cells^33^. The difference could be attributed to the different cell types and culture conditions used in both studies. Thus, further studies are required to analyse the dynamics of live cell viability and secretome production over time.

In our study, the neurotrophic effects mediated by DPSC-CM were associated with the presence of different levels of NGF, BDNF, GDNF and NT-3, which is in accordance with findings from other studies^22,32^. NGF, BDNF, and NT-3, support the survival of subpopulations of sensory neurons through their cognate Trk receptors. NGF was reported to promote corneal nerve regeneration and its blockage significantly impaired the promotion of neurite outgrowth^34^. Another study reported that the addition of different concentrations of exogenous NGF to neuronal cultures did not significantly influence the number of surviving neurons in vitro^35^. In this study, both NGF and DPSC-CM promoted cell viability and induced neurite outgrowth in comparison to those of serum-free culture, although the number of surviving neurons and the neurite length were significantly higher in DPSC-CM treated cultures. The advantage of CM over NGF treatment could be due to the presence of other soluble factors within CM, such as GDNF, BDNF, NT-3, and CNTF, which have also been proven to induce neurite outgrowth and promote neuronal survival in vitro^33,35,36^. These factors may act synergistically to promote neuronal survival and the development of neurite outgrowths.

Belzer et al. demonstrated that glial cells in adult rat TG culture showed a great reduction in glutamate synthase enzyme content after 2 days in culture. This enzyme is important for neuron-glial cell interactions; therefore, from day 2 onwards, the cells can no longer be considered genuine glial cells, eliminating out their trophic effects on the cultured neurons^37^. TGNC isolated in this study and grown in the absence of serum showed a great reduction in the number post isolation, as an axotomy of the three main branches of TG was required during the isolation process; thus, there was neuronal loss and profound phenotypic changes as a result of injury^38,39^. This effect was reversed when DPSC-CM was added as a linear trend of neuronal survival/rescuing and neurite outgrowth could be observed over time.

Pain receptors such as TRPV1 and TRPA1 have been proven to be upregulated by neurotrophins, especially NGF^35,40,41^. In this study, under the influence of DPSC-CM, TRPV1 mRNA gene expression was upregulated in TGNC. A dramatic upregulation of neuronal markers NeuN, MAP-2, and ßIII-tubulin in addition to synapsin-I was detected in the cultures treated with DPSC-CM. GAP-43 was expressed in neurons growing in vitro or in vivo, and the expression coincided with the beginning of neurite outgrowth^42^. In this study, GAP-43 was significantly upregulated in DPSC cultures compared with NGF treated cultures. These data suggest that soluble secreted factors from DPSC are able to stimulate relevant neural-associated gene expression in TGNC, increasing the expression of neuronal markers and axonal regeneration after cell isolation-induced injury.

## Conclusion

To the best of our knowledge, this is the first study to compare between the neuroprotective and neurogenic impact of DPSC and their CM on primary isolated TGNC. The results obtained indicate that TGNC survival and neurite outgrowth were significantly promoted by the concerted action of neurotrophic factors secreted by DPSC and collected in their CM and could be considered as a cell-free therapy for TG nerve repair.

## Data Availability

The datasets used and analysed in this study are available from the corresponding author on reasonable request.
